# Broken Ring enVision Search (BReViS): A New Clinical Test of Attention to Assess the Effect of Layout and Crowding on Visual Search

**DOI:** 10.3390/brainsci13030494

**Published:** 2023-03-15

**Authors:** Alessio Facchin, Maura Simioni, Silvio Maffioletti, Roberta Daini

**Affiliations:** 1 Department of Psychology, University of Milano-Bicocca, 20126 Milan, Italy; maura.simioni01@universitadipavia.it (M.S.); roberta.daini@unimib.it (R.D.); 2 COMiB—Optics and Optometry Research Center, University of Milano-Bicocca, 20126 Milan, Italy; 3 NeuroMI—Milan Center for Neuroscience, 20126 Milan, Italy; 4 Institute of Research and Studies in Optics and Optometry, 50059 Vinci, Italy; silvio.maffioletti@gmail.com; 5 Department of Brain and Behavioural Sciences, Università di Pavia, 27100 Pavia, Italy

**Keywords:** visual search, attention, neuropsychological tests, reference value, crowding

## Abstract

The assessment of attention in neuropsychological patients could be performed with visual search tests. The Broken Rings enVision Search test (BReViS) here proposed represents a novel open access paper-and-pencil tool in which layout and crowding are varied among four cards. These manipulations allow the assessment of different components of attention: a selective component, the visuo-spatial orientation of attention, and the focal attention, involved in a crowding phenomenon. Our purpose was to determine the characteristics of the BReViS test, provide specific normative data, and assess these components across the lifespan. The test was administered to a sample of 550 participants aged between 20 and 79 years old and to a series of patients. Three indexes targeting different components of visuo-spatial attention (selective attention, strategic orientation of visual attention, focal attention) were obtained by combining execution times and accuracy together with the total errors. The results showed that age, education and gender influenced, in different combinations, the four indexes, for which specific norms were developed. Regression-based norms were provided in percentiles and equivalent scores. All patients showed pathological scores and specific patterns of attentional deficits. The BreViS test proved to be a free and easy valuable tool which can be used in the clinical environment to assess attentional deficits in neuropsychological patients.

## 1. Introduction

During everyday life, a great amount of time is spent doing visual search tasks, without most people even realising it. Searching for a parking spot in a crowded place, looking for something to eat at the vending machines, examining the fruit at the supermarket while searching for a perfect apple: these are all different kinds of visual search tasks. Visual search has been a major topic of research since Helmholtz [1].

Visual search is not only an important real-world phenomenon, but also a very useful experimental paradigm for studying selective visual attention and its characteristics [2,3,4]. In classic laboratory search tasks, participants are asked to look for a target among a variable number of distractors and establish whether it is present or absent [5]. The difficulty of the task, and therefore, the amount of effort that has to be put to identify the target, depends on the number of features in common with distractors, the number of stimuli in the visual scene, i.e., “set size” [6], their arrangement in the visual scene [7,8] and the similarity between distractors and the target [9]. The different combination of these factors creates a continuum that ranges from “pop-out” searches, in which the target captures one’s attention directly, to “sequential searches”, in which the subject has to scan the visual scene element by element to foveate and localise the target [8]. Visual search tasks have often been used together with eye-tracking devices, which represent a useful tool for the study of human attention, allowing the tracking of shifts in attention through the recreation of the pattern of saccades and fixations carried out by the individual [10]. However, outside of the laboratory, eye-tracking devices are not practical and can be replaced by other measurement methods of oculo-motor behaviour [11,12,13].

Visual search tasks (VST) have also been used in clinical neuropsychology to evaluate selective visual attention impairments in neurological patients [14,15,16]. They usually consist of paper-and-pencil target cancellation tasks in which participants have to search for one or more targets among distractors and cancel them out using a pencil. Performance is usually evaluated by measuring execution time and accuracy. These kinds of tasks are useful for the evaluation of patients in which selective visual attention is commonly impaired, such as patients with traumatic brain injury [17], Alzheimer disease [18] and developmental dyslexia [19]. Visual search tasks are one of the most commonly used methods to assess spatial neglect [20,21], but in this specific application, the process of scoring is performed by comparing left and right omissions [22] or their single specific position [23].

The importance of normed VST for both adults and children arises from the fact that cancellation tasks are more sensitive than other attentional tasks at identifying age-related effects on attention [24] and they are valid instruments to detect attention deficit after stroke [25]. Indeed, attention and executive functions tend to improve during childhood and adolescence, reaching their maximum peak during adulthood. Subsequently, they decrease with ageing due to the modifications that attentional neural networks undergo during the lifespan [26,27].

Over the years, several VSTs have been proposed for clinical purposes. Excluding those specifically aimed at diagnosing spatial neglect [28], those proposed only for experimental purpose [29] and computerised tasks [30], the VSTs with specific norms in our knowledge are: the Attentional Matrices [31,32], the VSAT: Visual Search and Attention Test [33], the Ruff 2 & 7 Selective Attention Test [34], the letter cancellation test [35], the Multiple Features Target Cancellation [36], the Sustained Attention subtest in the Leiter-3 Battery [37] and the Visuo-spatial attention subtest in the Oxford Cognitive Screening [38].

The presence of different norms for adults and children allows the use of some tests throughout lifespan. However, there are few tools with normative data extended to different age phases [37], while others have been normed only for specific age ranges [31]. Moreover, they differ in their psychophysical characteristics, which have a direct impact on their difficulty level.

Existing VSTs do not take into account the potential effects of both stimuli’s layout and crowding on participants’ performance. When they do, indeed, they consider only one of the two dimensions [39,40]. Moreover, these tasks are frequently designed using different stimuli (e.g., letters, numbers, stars etc.) that require distinct visual and semantic processing, making it challenging to determine the effect of the arrangement of stimuli without confounding variables.

The Broken Rings enVision Search test (i.e., BReViS) here proposed represents a new alternative to the aforementioned classic paper-and-pencil visual search tests, allowing not only the assessment of selective visual attention, but also of its specific components, by changing the arrangement of stimuli in the visual scene.

In particular, the allocation of attentional resources in the visual space involves two distinct processes: an orientation process, which shifts the attentional resources to the relevant location for further processing [6,41], and a focusing process, which acts as a magnifying lens, allowing us to concentrate our resources selectively on a limited portion of the visual space and changing the spatial resolution according to the needs [42,43]. Moreover, the attention-resolution account argues that crowding reflects the limitation of the spatial resolution of attention [44,45,46]. As a result, closely grouped targets and distractors can be said to lead to impaired target discrimination because the resolution of attention is insufficient to disambiguate the relevant and irrelevant elements in the integration field [47]. Previous studies suggest that attention could reduce the size of the integration field responsible for crowding [48], and that the specific component of attention involved is the focal one [49]. Since the components of selective attention can therefore be selectively impaired [50], the new tool provides conditions that allow to isolate these components, by varying the layout and the crowding.

The BReViS consists of a four-cards cancellation task. In each card, 155 stimuli are displayed varying the layout (linear vs. random) and the level of crowding (high vs. low). The symbols used to create the matrices are Landolt rings, which is a standard optotype that consists of a ring with a gap, looking similar to the letter C. The target that has to be crossed out in each card is represented in the upper part of the sheet. This test gives the clinician the possibility to evaluate not only selective visual attention, but also the ability to strategically orient selective attention (by comparing the two possible layouts) and the ability to adapt the size of the focus of attention according to the disposition of the stimuli in the array (by comparing the two levels of crowding).

Additionally, it should be emphasised that the BReViS will be provided free of charge as an open access tool for professionals to assess visual search abilities without additional costs, removing limitations of test batteries or the difficulties of obtaining tests.

Due to the novelty of the BReViS and its potential utility in neuropsychological evaluations, the aim of the present study was to provide specific normative data, in order to obtain a useful diagnostic tool for the assessment of different attentional processes in the adult population.

## 2. Materials and Methods

### 2.1. Participants

A power analysis was performed to assess the minimum sample size required for defining regression based normative data. We based this analysis on the regression model with the following parameter: alpha = 0.05, power = 0.80, three predictors (demographic variables: age, education and gender) and a small effect size, f^2^ = 0.02. The result reports a minimum required sample size of 550 participants.

The test was administered to a group of native Italian healthy participants who had no current or past history of neurologic or psychiatric diseases (including brain injury, stroke, clinically diagnosed dementia, depression, alcohol or drug abuse), and achieved a normal score on the Mini-Mental State Examination (MMSE adjusted score > 23.8, [51]). All participants had normal or corrected-to-normal vision. Participants were recruited as a convenience sample among volunteers who could be directly contacted by the different examiners. No compensation was provided. A group of 563 healthy Italian participants was initially enrolled in the study. Due to the presence of extreme outliers in the performance time, 13 participants were excluded, leading to a final sample of 550 participants (see Section 2.4). The specific procedure used for filtering is the same as previously used [12] and is detailed in the statistical methods section.

**Figure 1:**
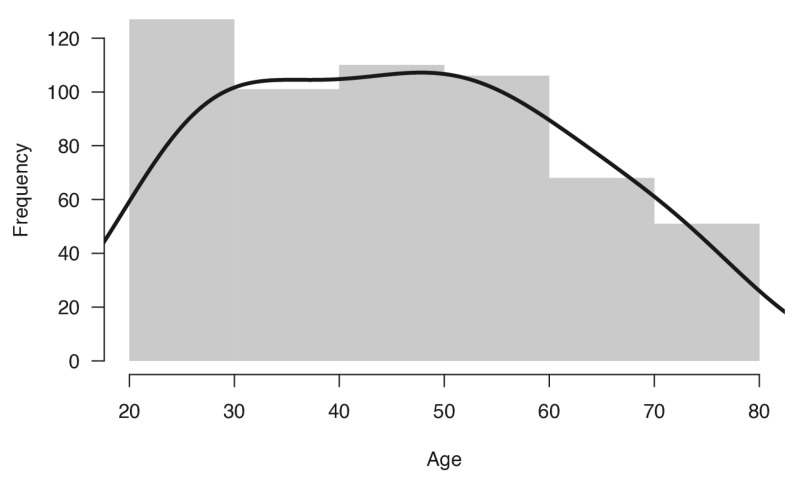
Frequency plot displaying the age distribution of the participants’ sample.

Therefore, the final sample included 550 healthy Italian volunteers (294 women and 256 men), distributed across age groups (age range, 20–79) and education levels, who took part in this study. Mean age of the sample was 45.53 years (SD 15.83) and mean formal education was 13.7 years (SD 4.0). The distribution of the sample for age and education is reported in Table 1 and displayed in Figure 1. Participants were divided into groups by decade of age. The distribution of sample between age groups, compared with the distribution of the adult Italian population in 2020 [52], was not significantly different (χ^2^_(5)_ = 3.01, *p* = 0.69).

The test was also administered to eight adults with Specific Learning Disabilities (SLD) recruited at University of Milano-Bicocca and to nine neurological patients recruited at the Neuropsychological Unit of the Sant’Antonio Abate Hospital, Somma Lombardo, Varese (Italy), who suffered focal right- (RBD) and left-brain damage (LBD) or underwent surgical neoplastic tumour resection (NTR). Demographic characteristics of the three patient groups are reported in Table 2.

Before the evaluation, the participants signed informed consent in order to participate in the study. The study was carried out following the guidelines given in the Declaration of Helsinki and it was approved by the Optics and Optometry Institutional Review Board of the University of Milano-Bicocca (prot. n. 5/2019; 13 May 2019).

### 2.2. The BReViS Test

The BReViS test consists of a four-card cancellation test aimed at assessing different attentional processes. It includes four subtests, each consisting of a standard A4 210 mm × 297 mm printable sheets, in which 155 stimuli are displayed varying the layout (linear (Cards 1 and 2) vs. random (Cards 3 and 4)) and the level of crowding (high [Cards 2 and 4) vs. low (Cards 1 and 3)]. The four cards thus obtained are:

(1) Linear layout, low crowded;

(2) Linear layout, high crowded;

(3) Random layout, low crowded;

(4) Random layout, high crowded.

**Figure 2:**
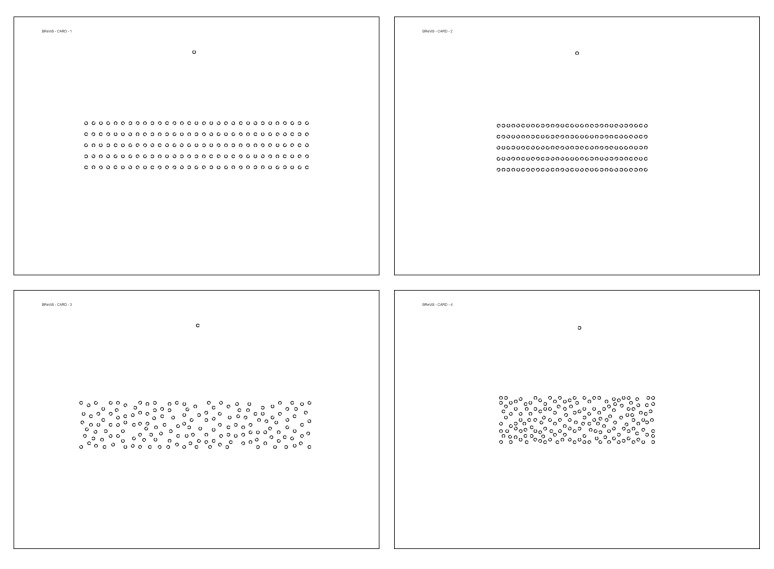
Scaled representation of the four cards of the BReViS test. (**Top left**), Card 1: Linear layout, low crowded; (**Top right**), Card2: linear layout, high crowded; (**Bottom left**), Card 3: Random layout, low crowded; (**Bottom right**), Card 4: Random layout, high crowded. All materials for administration and scoring of BReViS test are available online at https://osf.io/c64jg/ (accessed on 13 March 2023).

The four cards are displayed in Figure 2.

The symbols used to create the matrices are Landolt rings, which is a standard optotype commonly used to measure visual acuity [53]. It consists of a ring with a gap, looking similar to the letter C. The width of the C and its gap correspond to 1/5 of the diameter of the ring, as indicated in the UNI EN ISO 8596:2017 [54].

In each matrix, the gap of the Landolt rings was placed in the eight possible positions: four are vertical or horizontal at 0°, 90°, 180° and 270°, and four diagonally at 45°, 135°, 225° and 315°. In the subtests with linear arrangement the Landolt rings are displayed in 5 rows and 11 columns. The stimuli in the random arrangement subtests are distributed randomly within the same inter-stimulus space of linear layout. Symbol matrices are centred on an A4 sheet. Matrix size is 184 mm × 39 mm for the low crowded condition and 123 mm × 39 mm for the high crowded condition. Moreover, in linear crowded matrices the horizontal distance between the stimuli is 1 mm, while in linear uncrowded matrices, the horizontal distance is 3 mm. The size of the target is 3 mm, which at 40 cm distance corresponds to a visual acuity of 0.2 (decimal) or +0.7 LogMAR.

The target that has to be found in each subtest is represented in the upper part of each sheet and has a gap in one out of the four vertical and horizontal positions (Card 1: up; Card 2: down; Card 3: right; Card 4: left). The number of targets that had to be identified in each subtest is 25. The position of targets was defined randomly. A single pre-test card in which four small matrices with similar characteristics to the four test cards was created and it had the purpose of familiarising participants with the task. Four transparency sheets for the evaluation of the test were created, and they could be superimposed on each card in order to evaluate the errors performed (see below). The test is released in open source and open access form with no restrictions on its use. All materials (test cards, transparency sheets and scoresheet) are freely available at https://osf.io/c64jg/ (accessed on 13 March 2023).

The instructions verbally given were:

“This test evaluates how accurate you are in finding some symbols among others. There are five rows with many rings, which have a gap in different possible directions. Your aim is to find and cancel all the circles that have the gap in the same direction as the symbol displayed at the top of the page. You should try to be both fast and accurate. If you realise that you have made a mistake, please do not go back but proceed until the end. When you have finished, place the pen on the table.”

Execution time, number of omissions (targets not crossed), number of autocorrections and number of substitutions (incorrect symbols crossed) were recorded.

In the evaluation of performance of visual search tasks, the execution time is sometimes adjusted for the number of errors performed. A variety of methods were proposed [55,56,57,58], and the BReViS test scoring also follows this criterion using a specific formula [11,59]. Performance time for each card was calculated combining execution time and omissions (in the same card) with the following formula:

$$\text{Performance}\;\text{time}=\frac{25\times \text{Execution}\;\text{time}}{25-\text{Omissions}}$$

Four indexes were calculated as follows combining the performance time obtained in the four cards.

(1) Selective Attention (SA)

This index represents the ability to suppress irrelevant stimuli (distractors) and select only relevant stimuli (targets) in the easiest condition. It corresponds directly to the performance time of the first Card (linear layout, low crowded), which is less influenced by a random array and a crowded display. High values on this index should be interpreted as indicative of less efficient selective attention.

(2) Orientation of Attention (OA)

This index refers to the Strategic Orientation of Visual Attention, such as the ability to direct visuo-spatial attention throughout the visual scene following an effective endogenous strategy. This is independent of the crowding of the stimuli in the array. It corresponds to the comparison of the two levels of layouts: linear and random. High values on this index indicate the inability to follow an effective endogenous strategy during visual search and the need for exogenous cues to perform correctly the task. It is calculated with the following formula using the performance time of each card:

$$OA=\frac{Card3+Card4}{2}-\frac{Card1+Card2}{2}$$

(3) Focal Attention (FA)

This index can be interpreted as the ability to adapt the size of the focus of attention according to the disposition of the stimuli in the array, and it corresponds to the comparison between the two levels of crowding: high and low. High values on this index indicate a higher susceptibility to crowding, while lower values are the result of an efficient modulation of the focus of attention. It is calculated with the following formula using the performance time of each card:

$$FA=\frac{Card2+Card4}{2}-\frac{Card1+Card3}{2}$$

(4) Total Errors (Err)

This index reflects the overall errors performed in all subtests. It is calculated with the sum of all errors performed in all cards.

### 2.3. Procedures

The evaluation was carried out in a lab or in a quiet and well-illuminated dedicated room (>350 lux). Before the beginning of the assessment, each participant was requested to sign an informed consent and the experimenter checked the inclusion and exclusion criteria. Participants were seated at a desk wearing the correct glasses (if necessary) and each card was placed at a distance of approximately 40 cm, aligned with the mid-sagittal plane. Each of the four cards was preceded by a run-in pre-test to familiarise participants with the task (pretest1, card 1, pretest2 and card2, etc.) and to assess the presence of the minimal cognitive and visual-acuity requirements needed to perform the task. A stopwatch was used to record execution time. The four cards were presented in the following order: (1) Linear layout, low crowded; (2) Linear layout, high crowded; (3) Random layout, low crowded; (4) Random layout, high crowded. Before the beginning of each subtest, the investigator showed the specific target, located at the top of the page, to the participant. They were verbally instructed to cross out all the targets in the shortest amount of time. Timing began when the participant picked up the pen and stopped when the participant laid the pen down on the table. All participants performed all tasks in a single session. Other staff members could be present in the lab/room, but they did not interact with the participants. The whole experiment took about 15 min. All conditions tested and all dependent variables were reported. Specific instructions can be found in the scoresheet available at https://osf.io/c64jg/ (accessed on 13 March 2023).

### 2.4. Statistical Analysis

While the instructions given clearly state that the pen should be put on the table once the visual search was completed, some participants continued and/or repeated the visual search to identify errors, increasing the execution time. To overcome this problem, a posteriori case-wise removal of the univariate higher extreme outliers was performed. Based on the performance time of each card, the non-parametric threshold for the identification of extreme outliers was calculated as three times the interquartile range (3 × IQR) over the t quartile [60]. According to the performance time, the threshold values obtained were the following: 144.5 s for Card1, 144.6 s for Card2, 230.5 s for Card 3 and 304.6 s for Card4. If the performance time of at least one card was equal or greater than the non-parametric thresholds, all data from that individual were discarded. With this procedure, a total of 13 participants’ data were deleted, reducing the final sample from 563 to 550 participants.

The analyses were then divided into three parts. In the first part, more descriptive, the comparison between indices of attention and age was performed. An ANOVA using a Wald test with the independent variable, Age, was carried out for each index. The descriptive data may be useful for the calculation of a z-score when comparing different tests and can show the trend of attention in the lifespan. Correlation between demographic variables and BReViS subtests were performed using Pearson or point biserial correlations.

Normative reference values were defined in the second part. The regression-based procedure definition of norms was used, which includes several consecutive steps [12,61,62,63] in line with other visuo-spatial [64,65] and different neuropsychological tests [66,67]. First, in order to find the most appropriate transformation of confounding demographic variables (age, education and gender) on the dependent variables (the four indexes mentioned above, taken separately), the general linear model (GLM) was used. A series of bivariate regressions were compared based on the lowest Akaike’s Information Criterion (AIC) [68,69]. The most effective transformations of demographic independent variables were selected, and they were included in a series of bivariate and multivariate regressions with one to three predictors for a total of seven models. Based on the smaller AIC, the most appropriate regression model from the set of seven was selected, if it was significant (*p* < 0.05). The same best regression model was applied to deviations from the mean transformed scores for independent variables (in their appropriate transformations) and dependent variables. Reversing the coefficients of the last regression, a correction regression equation was then obtained. Based on the correction regression, a grid for each score was created, with the aim of facilitating the scoring process during clinical practice. However, to improve the precision of the scoring, the correction regression equations should be used. Adjusted times for demographic variables were obtained, adding the correction score to the raw performance times. On these adjusted times, one-sided parametric or nonparametric 95% tolerance limits (depending on distribution), with a confidence interval of 95%, were calculated. Subsequently, percentile ranks and equivalent scores [61] on the adjusted time were calculated with a rank method [70].

Finally, using the normative data obtained, the evaluation of patients was reported. Their specific scores were calculated and reported in a table. Data were analysed using R statistical environment and specific packages [71].

## 3. Results

### 3.1. Descriptive Results

The descriptive mean results on the four BReViS scores are reported in Table 3.

**Figure 3:**
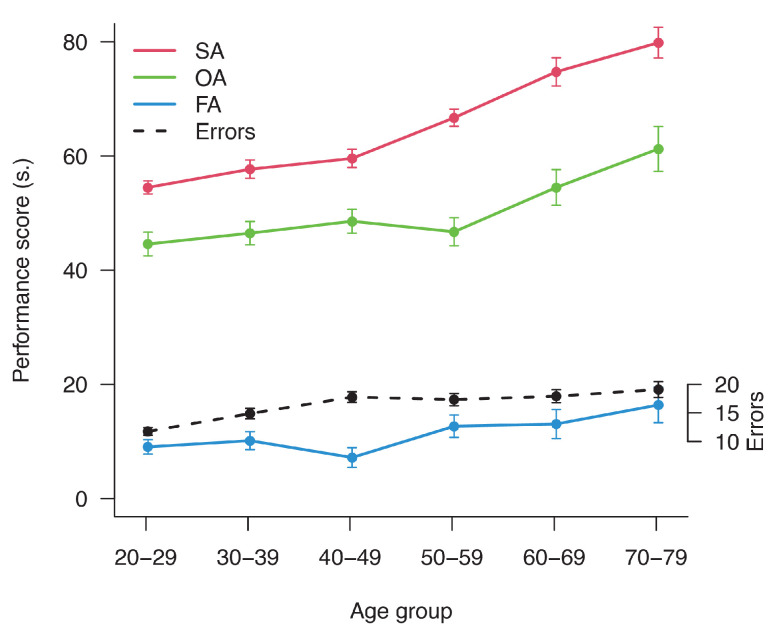
Descriptive means plot of the 4 different indexes of BreVis test separated for age groups: SA, OA, FA and total errors. Bars represent +/− 1 SEM.

The results of the ANOVA showed a significant effect of Age in SA [Χ^2^_(1)_ = 133.12 *p* < 0.001], OA [Χ^2^_(1)_ = 18.46 *p* < 0.001], FA [Χ^2^_(1)_ = 8.59 *p* < 0.005] and Errors [Χ^2^_(1)_ = 31.11 *p* < 0.001]. Results are displayed in Figure 3, and they show a general increment of performance time and errors with age. 

**Figure 4:**
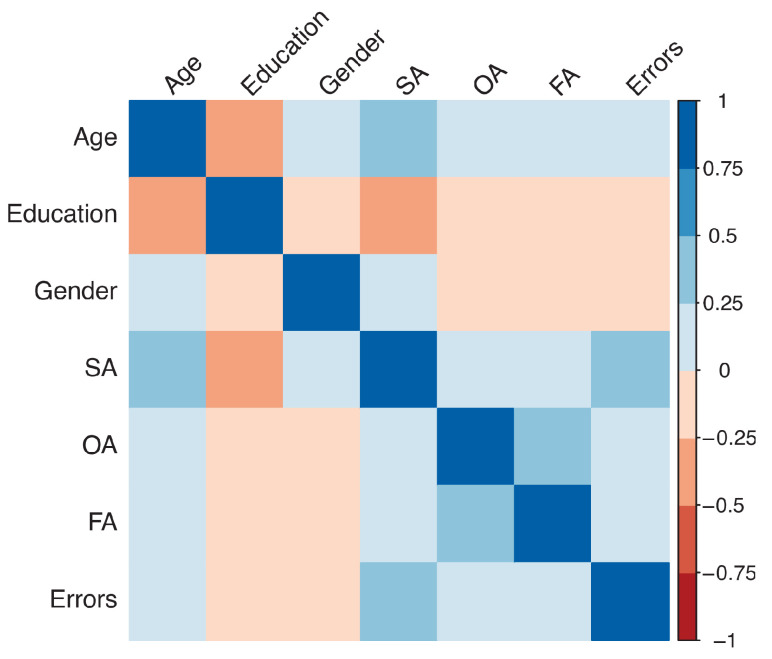
Correlation plot of the demographic variables and the four different indexes of the BreVis test.

The correlation between demographic characteristics and the BreVis indexes are displayed using the correlation plot in Figure 4. Results showed a positive moderate correlation between Age and SA (r = 0.44, *p* < 0.001) and a low to moderate negative correlation between Education and SA (r = −0.35 *p* < 0.001). In addition, a point biserial correlation between gender and BReViS indexes showed only a negative low correlation between gender and the total number of errors (r_pb_ = −0.13 *p* < 0.001).

### 3.2. Definition of Normative Values

The bivariate and multivariate regressions selection with the AIC method showed that the model that better describes the Selective Attention index includes age and education, but not gender in their respective transformations (inverse additive logarithmic and inverse). The models for the Strategic Orientation of Visual Attention index and the Focal Attention index, instead, include only age in a cubic transformation. Total Errors includes age, education in an inverse transformation and gender. AIC tables used for choosing the best models are visible in Table 4. For FA, since the difference between the models that include age and those that include age and gender was very small, the simplest model was selected.

The multiple regressions were then redrawn from deviation from the mean score, in order to obtain correction values, and they are reported in Table 5. To allow a faster clinical application, correction grids were constructed from these regression equations and are available in Table 6, Table 7 and Table 8 for a straightforward correction.

All the adjusted times were not normally distributed (all *p* values < 0.001), consequently, the one-side inner and outer 95% tolerance limits with 95% confidence intervals were calculated using a non-parametrical approach. For a sample of 550 participants, using a score in which the lower, the better, they correspond to the 515th and 532nd ordered observations. They can be found in Table 9 together with borderline scores.

The cut-off scores provided by the outer tolerance limits, together with median and other intermediate intervals were subsequently transformed into rank-based Equivalent Scores (ES). Moreover, for each index, percentiles were calculated. They are available in Table 10 and Table 11, respectively.

### 3.3. Example of BReViS’s Application in Patients

The BReViS test was applied to different sets of patients examined. The number of patients who obtained a pathological score separately for each index and patient’s diagnosis was reported in Table 12. The scores were considered pathologic if they fell in the 0 ES areas, and in accordance with the detailed procedure, they were corrected for demographic variables.

Even in this small and varied sample of patients, the BReViS test has permitted to show specific impairment in different forms of attention. As expected, the RBD group exhibited the greatest alteration in total accuracy and the three different attentional indexes; moreover, some LBD patients, some adults with specific learning disorder and the single patient who underwent surgical tumour resection showed alterations, mainly in selective attention.

## 4. Discussion

The primary aim of the present study was to define the characteristics of a new neuropsychological tool for the assessment of attention and its components, the BReViS test, and provide specific normative data. A secondary aim was to investigate the effect of Crowding and Layout on visual search in the lifespan.

The BReViS test comprises four different indexes that can be used for the evaluation of the patients’ performance, which measure different components of visual attention. First of all, similarly to other VSTs, the BReViS was created for the evaluation of selective attention (SA), which is the ability to select part of simultaneous sources of information by enhancing the processing of the objects of interest and suppressing the distracting information [72]. The results showed that the SA index is influenced by both age and education, in line with the results of previous studies [26,55,73].

Two components of visuo-spatial attention can be measured with the aid of the BReViS: the Strategic Orientation of Visual Attention (OA), which refers to the ability to direct selective visual attention throughout the visual scene, following an effective endogenous strategy [74,75] and the Focal Attention (FA), which indicates the ability to adapt the size of the focus of attention according to the proximity of the stimuli in the array [42,76]. By looking at the statistical model in the normal population, it is possible to see that SA index is more sensitive to detect age differences compared to OA, FA and errors. In other words, the changes linked to age are larger for SA. Orientation of attention, in turn, measuring the efficiency of the deployment of attention throughout the visual array, could be influenced by the reduction in the efficiency and speed of oculo-motor movements that has been documented in both pathological and normal aging [77]. Albonico et al. [49], indeed, showed that the critical distance (i.e., the inter-stimulus distance needed to avoid the effect of crowding) is influenced by focal attention and Daini et al. [50] found a link between focal attention and crowding in right brain-damaged patients. Specifically, they found that patients more sensitive to crowding (i.e., who did more letter identification errors in unspaced conditions and improved their performance with spaced stimuli) also showed a deficit of focal attention. Here, we found that the two indexes of visuo-spatial attention, as expected in light of previous studies [78], are influenced by age. Finally, the Total Errors index, which takes into account all the errors made in the four cards, is influenced by Age, Gender and Education. The inclusion of this index alongside the others gives the possibility to distinguish between defective performances due to low accuracy or to a general ideomotor slowing.

A key component of the BReViS test is the consideration that crowding and layout may impact visual search differently according to different mechanisms. None of the other VSTs, indeed, take into account both aspects as potential interfering factors, so that in order to test their effect it was necessary to use more than one test. However, the use of two VSTs that differ not only for the arrangement of stimuli but also for other variables makes the comparison more confusing. For instance, different VSTs are usually built using a variety of symbols (e.g., letters, numbers, etc.), which require different semantic processing. This introduces a source of variability that makes it risky to compare participants’ performance. The BReViS, thanks to the use of the Landolt ring, a standardised meaningless symbol commonly used for the measurement of visual acuity, overcomes this inconvenience and allows to study the effects of layout and crowding keeping all the other visual features constant.

The normative data provided here permit the assessment of the different components of attention in a clinical population simply using the test available online and the scoring system outlined above.

Due to the fact that the participants enrolled in this study were Italian, the norms could be correctly classified as Italian norms. Nevertheless, since ethnicity has no influence on VSTs [55], as in many visuo-spatial tasks, these norms can be used as an independent international reference. For a broad application, since adjusted scores were not normally distributed, both percentile and equivalent score were reported.

Observing the test, a limitation in face validity seems to arise from the structure of the matrix. Indeed, the two spacing conditions were not large and small enough to assess crowding at its best, but seem two moderate sizes. Despite this, the effect of crowding emerges clearly and if we compare stimuli in the real world, as much as book or journal content, the low crowded condition represents the distance between words and the high crowded condition the inter-letter distance within words.

It is well established in the literature and clinical practice that VSTs are valid tests to measure selective attention [24]. BReViS offers a measure of selective attention, but also one of strategic orientation of visual attention and another one of focal attention. However, in the small group of patients tested, a dissociation between the four indexes was recorded and it can be considered a simple form of predictive validity. Moreover, some clinical vs. tests do not have a specific assessment of validity other than the one that compares neurological patients to healthy controls [79], as we did. In fact, in the small group of patients assessed with the BReViS, the performance is below the cut-off in each one of the scores proposed. The RBD patients, as expected, showed more deficits than the other groups, confirming the validity and the applicability of BReViS [80].

Inevitably, some aspects remain open, such as specific validity of some subscores as well as other normative data for specific populations (developmental ages and unilateral spatial neglect patients). These issues may be addressed in future studies taking into account large groups of patients with different aetiology or focal lesions.

The BReViS test is provided in the online Supplementary Materials, without restriction of its use (open source and open access). As a result, different professionals interested in studying and clinically assessing visual search will have an easy-to-print tool.

## Figures and Tables

**Table 1:** Demographic characteristics of the participants’ sample. F = female; M = male.

Age	20–29		30–39		40–49		50–59		60–69		70–79		Tot.
School	F	M	F	M	F	M	F	M	F	M	F	M	
0–5	0	0	0	0	0	1	0	0	0	3	6	2	12
6–8	0	0	2	5	3	5	3	9	9	6	10	10	62
9–13	11	12	16	20	25	21	34	22	19	20	4	10	214
>13	55	30	34	32	33	19	22	15	5	9	3	5	262
Tot.	66	42	52	57	61	46	59	46	33	38	23	27	550

**Table 2:** Demographic characteristics of the patients’ sample. ALD = Adult developmental learning disability patients; RBD = Right Brain Damaged patients; LBD = Left Brain Damaged patients; NTR = post neoplastic tumour resection patient.

Group	N	Sex	Mean Age (SD)	Mean Education (SD)
ALD	8	8F	21.5 (2.1)	14.9 (1.2)
RBD	3	2F-1M	68.7 (11.9)	13.3 (5.5)
LBD	5	1F-4M	64.2 (9.0)	13.2 (5.7)
NTR	1	1F	63	8

**Table 3:** Mean performance time (and SD) for each subtest, divided by age group.

Subtest	20–29	30–39	40–49	50–59	60–69	70–79
SA	54.5 (11.8)	57.7 (16.8)	59.6 (16.5)	66.7 (15.4)	74.8 (20.8)	79.9 (19.0)
OA	46.5 (21.5)	48.6 (21–4)	46.7 (21.5)	44.6 (25.1)	54.5 (26.4)	61.2 (27.6)
FA	9.0 (13–1)	10.1 (16.3)	7.2 (17.9	12.7 (20.1)	13.1 (21.4)	16.4 (22.0)
Err	11.8 (6.9)	14.9 (9.6)	17.8 (9.7)	17.3 (11.0)	17.9 (9.6)	19.1 (9.8)

**Table 4:** Comparison between regression models aimed at finding the best transformation of independent variables for each BReViS subtest. SA = Sustained attention; OA = Orientation of attention; FA = Focus of attention; Errors = Total number of errors; K = Number of parameters of the model; AICc = Akaike’s Information Criterion corrected; Delta AIC = AIC difference between the best model and the model listed; Model Lik. = the relative likelihood of the model; AICc Wt = model probabilities; LL = log-likelihood of the model; Cum. Wt = cumulative Akaike weights.

	Models	K	AICc	Delta_AICc	ModelLik	AICcWt	LL	Cum.Wt
SA	Age + Edu	4	4616.249	0	1	0.731	−2304.09	0.731
	Age + Edu + Sex	5	4618.245	1.995	0.369	0.269	−2304.07	1
	Age	3	4638.388	22.138	0	0	−2316.17	1
	Age + Sex	4	4640.41	24.16	0	0	−2316.17	1
	Edu	3	4681.157	64.907	0	0	−2337.56	1
	Edu + Sex	4	4683.142	66.892	0	0	−2337.53	1
	Sex	3	4763.543	147.294	0	0	−2378.75	1
OA	Age	3	5031.837	0	1	0.487	−2512.9	0.487
	Age + Edu	4	5033.305	1.468	0.48	0.234	−2512.62	0.72
	Age + Sex	4	5033.721	1.884	0.39	0.19	−2512.82	0.91
	Age + Edu + Sex	5	5035.21	3.373	0.185	0.09	−2512.55	1
	Edu	3	5052.412	20.575	0	0	−2523.18	1
	Edu + Sex	4	5054.423	22.586	0	0	−2523.17	1
	Sex	3	5054.875	23.038	0	0	−2524.42	1
FA	Age + Sex	4	4750.881	0	1	0.305	−2371.4	0.305
	Age	3	4750.967	0.086	0.958	0.292	−2372.46	0.597
	Age + Edu + Sex	5	4751.845	0.964	0.618	0.188	−2370.87	0.785
	Age + Edu	4	4752.079	1.198	0.549	0.167	−2372	0.952
	Edu	3	4756.205	5.324	0,07	0.021	−2375.08	0.974
	Edu + Sex	4	4756.308	5.427	0.066	0.02	−2374.12	0.994
	Sex	3	4758.718	7.837	0.02	0.006	−2376.34	1
Errors	Age + Edu + Sex	5	4021.139	0	1	0.834	−2005.51	0.834
	Age + Sex	4	4024.424	3.285	0.193	0.161	−2008.18	0.995
	Age + Edu	4	4031.78	10.641	0.005	0.004	−2011.85	0.999
	Age	3	4034.46	13.321	0.001	0.001	−2014.21	1
	Edu + Sex	4	4041.586	20.447	0	0	−2016.76	1
	Edu	3	4050.334	29.195	0	0	−2022.14	1
	Sex	3	4058.436	37.297	0	0	−2026.2	1

**Table 5:** Correction regressions aimed to obtain the correction score for the four indexes of BReViS. The adjusted time can be calculated by adding the raw performance time to the regression results. Gender was scored as 0 = female, 1 = man. The logarithms were intended to be computed on the natural base *e*.

Index	Regression for Obtaining Correction Score	R2	Adj. R2	RSE
**SA**	13.796 × (log(86.9 − Age) − 3.628) − 129.5 × ((1/Education) − 0.081)	0.238	0.236	16.01
**OA**	−0.00004 × ((Eta3) − 129295)	0.041	0.039	23.38
**FA**	−0.000019 × ((Eta3) − 129295)	0.017	0.015	18.11
**Err**	195.11 × ((1/Age) − 0.0251) − 38.13 × ((1/Education) − 0.0812) + 2.6 × (Gender − 0.47)	0.091	0.086	8.83

**Table 6:** Correction grid for computing the adjusted time for the Selective Attention (SA) index. The adjusted time can be calculated by adding the raw performance time to the reported value obtained by the table. Age (divided in 12 levels) and education (5 levels) should be selected based on the nearest values. If precise scoring is required, the correction regression should be used.

	Education				
Age	8	13	16	18	21
22	1.8	8	9.9	10.8	11.8
27	0.7	6.9	8.8	9.7	10.7
32	−0.5	5.7	7.6	8.5	9.5
37	−1.8	4.4	6.3	7.2	8.2
42	−3.3	3	4.8	5.7	6.8
47	−4.9	1.3	3.2	4.1	5.1
52	−6.7	−0.5	1.4	2.3	3.3
57	−8.9	−2.6	−0.8	0.1	1.1
62	−11.4	−5.2	−3.3	−2.4	−1.4
67	−14.5	−8.3	−6.4	−5.5	−4.5
72	−18.5	−12.3	−10.4	−9.5	−8.5
77	−24.1	−17.9	−16	−15.1	−14.1

**Table 7:** Correction grid for computing the adjusted time for Orientation of Attention (OA) and Focal Attention (FA). The adjusted time can be calculated by adding the raw performance time to the reported value obtained by the table. Age should be selected based on the nearest values. If precise scoring is required, the correction regression should be used.

Age	OA Correction Values	FA Correction Values
22	4.7	2.3
27	4.4	2.1
32	3.9	1.8
37	3.1	1.5
42	2.2	1
47	1	0.5
52	−0.5	−0.2
57	−2.2	−1.1
62	−4.4	−2.1
67	−6.9	−3.3
72	−9.8	−4.6
77	−13.1	−6.2

**Table 8:** Correction grid for computing the adjusted score for Total Errors (Err). The adjusted score can be calculated by adding the raw score to the reported value obtained by the table. Age and education should be selected based on the nearest values. If precise scoring is required, the correction regression should be used.

Education	Female					Male				
Age	8	13	16	18	21	8	13	16	18	21
22	1.1	2.9	3.5	3.7	4	3.7	5.5	6.1	6.3	6.6
27	−0.6	1.3	1.8	2.1	2.4	2	3.9	4.4	4.7	5
32	−1.7	0.1	0.7	1	1.3	0.9	2.7	3.3	3.6	3.9
37	−2.5	−0.7	−0.1	0.1	0.4	0.1	1.9	2.5	2.7	3
42	−3.1	−1.3	−0.8	−0.5	−0.2	−0.5	1.3	1.8	2.1	2.4
47	−3.6	−1.8	−1.3	−1	−0.7	−1	0.8	1.3	1.6	1.9
52	−4	−2.2	−1.7	−1.4	−1.1	−1.4	0.4	0.9	1.2	1.5
57	−4.4	−2.5	−2	−1.7	−1.4	−1.8	0.1	0.6	0.9	1.2
62	−4.6	−2.8	−2.3	−2	−1.7	−2	−0.2	0.3	0.6	0.9
67	−4.9	−3	−2.5	−2.2	−1.9	−2.3	−0.4	0.1	0.4	0.7
72	−5.1	−3.2	−2.7	−2.4	−2.1	−2.5	−0.6	−0.1	0.2	0.5
77	−5.3	−3.4	−2.9	−2.6	−2.3	−2.7	−0.8	−0.3	0	0.3

**Table 9:** Non-parametric inner (ITL) and outer (OTL) tolerance limits 95% tolerance limits with 95% confidence intervals.

Index	ITL	Borderline Scores	OTL
SA	90	90.1–97.3	97.4
OA	86.1	86.2–98.7	98.8
FA	41.0	41.1–49.1	49.2
Err	29.9	30.0–35.1	35.2

**Table 10:** Equivalent Scores for adjusted values for the four indexes of the BReViS test.

Equivalent Score	0	1	2	3	4
SA	≥97.4	97.3–74.8	74.7–67.1	67.0–61.5	<61.5
OA	≥98.9	98.8–67.9	67.8–55.6	55.5–46.3	<46.3
FA	≥49.2	49.1–24.8	24.7–15.5	15.4–8.4	<8.4
Err	≥35.2	35.1–23.1	23.0–18.1	18.0–14.7	<14.7

**Table 11:** Percentiles score for the four indexes of the BReViS test.

Percentiles	SA	OA	FA	Err
99	38.4	4.5	−29.3	1.0
95	42.2	12.9	−13.7	4.3
90	45.5	22.2	−8.8	6.1
85	47.6	27.2	−4.9	7.4
80	50.7	30.2	−2.7	8.3
75	52.5	34.1	−0.5	9.9
70	54	36.4	1.4	10.9
65	55.8	38.2	2.9	11.9
60	57.4	41.3	4.9	12.9
55	59.9	44.0	6.4	13.7
50	61.5	46.2	8.4	14.7
45	63.1	48.6	10.7	15.9
40	65	51.5	13.5	16.7
35	67.1	55.1	15.4	18.0
30	69.7	58.4	17.3	19.2
25	71.6	61.0	19.6	20.3
20	74	66.7	23.6	22.6
15	77.8	72.9	27.4	24.3
10	83.7	78.9	32.8	27.5
5	92.5	88.5	44.1	31.3
4	96.9	95.1	45.6	33.5
3	98	100.8	50.9	35.5
2	102.1	107.7	56.5	38.1
1	114.1	120.3	60.5	45.0

**Table 12:** Performance to the BReViS test in terms of pathological scores of patients classified by their clinical diagnosis. * = over OTL (0/1 equivalent score). ALD = Adult developmental learning disability patients; RBD = Right Brain Damaged patients; LBD = Left Brain Damaged patients; NTR = neoplastic tumour resection patient.

		n. of Pathological Scores * (%)			
Diagnosis	N	SA	OA	FA	Err
ALD	8	4 (50%)	2 (25%)	3 (37.50%)	2 (25%)
RBD	3	2 (66.66%)	2 (66.66%)	3 (100%)	1 (33.33%)
LBD	5	4 (80%)	3 (60%)	2 (40%)	0 (0%)
NTR	1	1 (100%)	0 (0%)	0 (0%)	1 (100%)

## Data Availability

The data presented in this study are available on request from the corresponding author. The data are not publicly available due to restrictions included in the informed consent provided by participants.

## Supplementary Materials

Test cards, transparency sheets and scoresheet are available at: https://osf.io/c64jg/ (accessed on 13 March 2023).

## References

[1] von Helmholtz H. (1903). Treatise on Physiological Optics.

[2] Bacon W.F., Egeth H.E. (1997). Goal-Directed Guidance of Attention: Evidence from Conjunctive Visual Search. J. Exp. Psychol. Hum. Percept. Perform..

[3] Verghese P. (2001). Visual Search and Attention: A Signal Detection Theory Approach. Neuron.

[4] Wolfe J.M. (2003). Moving towards Solutions to Some Enduring Controversies in Visual Search. Trends Cogn. Sci..

[5] Treisman A.M., Gelade G. (1980). A Feature-Integration Theory of Attention. Cognit. Psychol..

[6] Wolfe J.M., Horowitz T.S. (2017). Five Factors That Guide Attention in Visual Search. Nat. Hum. Behav..

[7] Gheri C., Morgan M.J., Solomon J.A. (2007). The Relationship between Search Efficiency and Crowding. Perception.

[8] Wolfe J.M. (2021). Guided Search 6.0: An Updated Model of Visual Search. Psychon. Bull. Rev..

[9] Duncan J., Humphreys G.W. (1989). Visual Search and Stimulus Similarity. Psychol. Rev..

[10] Holmqvist K., Nyström M., Andersson R., Dewhurst R., Jarodzka H., van de Weijer J. (2011). Eye Tracking: A Comprehensive Guide to Methods and Measures.

[11] Facchin A. (2021). Spotlight on the Developmental Eye Movement (DEM) Test. Clin. Optom..

[12] Facchin A., Mischi E., Iannello C., Maffioletti S., Daini R. (2022). Normative Values of the Groffman Visual Tracing Test for the Assessment of Oculomotor Performance in the Adult Population. Vision.

[13] Matzen L.E., Stites M.C., Gastelum Z. (2021). Studying Visual Search without an Eye Tracker: An Assessment of Artificial Foveation. Cogn. Res. Princ. Implic..

[14] Eglin M., Robertson L.C., Knight R.T. (1989). Visual Search Performance in the Neglect Syndrome. J. Cogn. Neurosci..

[15] Luck S.J., Hillyard S.A., Mangun G.R., Gazzaniga M.S. (1989). Independent Hemispheric Attentional Systems Mediate Visual Search in Split-Brain Patients. Nature.

[16] Utz K.S., Hankeln T.M.A., Jung L., Lämmer A., Waschbisch A., Lee D.-H., Linker R.A., Schenk T. (2013). Visual Search as a Tool for a Quick and Reliable Assessment of Cognitive Functions in Patients with Multiple Sclerosis. PLoS ONE.

[17] Schmitter-Edgecombe M., Robertson K. (2015). Recovery of Visual Search Following Moderate to Severe Traumatic Brain Injury. J. Clin. Exp. Neuropsychol..

[18] Tales A., Butler S.R., Fossey J., Gilchrist I.D., Jones R.W., Troscianko T. (2002). Visual Search in Alzheimer’s Disease: A Deficiency in Processing Conjunctions of Features. Neuropsychologia.

[19] Sireteanu R., Goebel C., Goertz R., Werner I., Nalewajko M., Thiel A. (2008). Impaired Serial Visual Search in Children with Developmental Dyslexia. Ann. N. Y. Acad. Sci..

[20] Ferber S., Karnath H.-O. (2001). How to Assess Spatial Neglect—Line Bisection or Cancellation Tasks?. J. Clin. Exp. Neuropsychol..

[21] Williams L.J., Kernot J., Hillier S.L., Loetscher T. (2021). Spatial Neglect Subtypes, Definitions and Assessment Tools: A Scoping Review. Front. Neurol..

[22] Evald L., Wilms I., Nordfang M. (2021). Assessment of Spatial Neglect in Clinical Practice: A Nationwide Survey. Neuropsychol. Rehabil..

[23] Rorden C., Karnath H.-O. (2010). A Simple Measure of Neglect Severity. Neuropsychologia.

[24] Vakil E., Blachstein H., Sheinman M., Greenstein Y. (2009). Developmental Changes in Attention Tests Norms: Implications for the Structure of Attention. Child Neuropsychol..

[25] Goldstein G., Welch R.B., Rennick P.M., Shelly C.H. (1973). The Validity of a Visual Searching Task as an Indicator of Brain Damage. J. Consult. Clin. Psychol..

[26] Plude D.J., Enns J.T., Brodeur D. (1994). The Development of Selective Attention: A Life-Span Overview. Acta Psychol..

[27] Trick L.M., Enns J.T. (1998). Lifespan Changes in Attention: The Visual Search Task. Cogn. Dev..

[28] Wilson B., Cockburn J., Halligan P. (1987). Development of a Behavioral Test of Visuospatial Neglect. Arch. Phys. Med. Rehabil..

[29] Faria A.L., Paulino T., I Badia S.B. Comparing Adaptive Cognitive Training in Virtual Reality and Paper-Pencil in a Sample of Stroke Patients. Proceedings of the 2019 International Conference on Virtual Rehabilitation (ICVR).

[30] Wang T.-Y., Huang H.-C., Huang H.-S. (2006). Design and Implementation of Cancellation Tasks for Visual Search Strategies and Visual Attention in School Children. Comput. Educ..

[31] Della Sala S., Laiacona M., Spinnler H., Ubezio C. (1992). A Cancellation Test: Its Reliability in Assessing Attentional Deficits in Alzheimer’s Disease. Psychol. Med..

[32] Spinnler H., Tognoni G. (1987). Standardizzazione e taratura italiana di test neuropsicologici: Gruppo italiano per lo studio neuropsicologico dell’invecchiamento. Ital. J. Neurol. Sci..

[33] Trennery M.R., Crosson B., DeBoe J., Leber W.R. (1990). Visual Search and Attention Test (VSAT).

[34] Ruff R.M., Allen C.C. (1996). Ruff 2 & 7 Selective Attention Test Professional Manual.

[35] Uttl B., Pilkenton-Taylor C. (2001). Letter Cancellation Performance Across the Adult Life Span. Clin. Neuropsychol..

[36] Marra C., Gainotti G., Scaricamazza E., Piccininni C., Ferraccioli M., Quaranta D. (2012). The Multiple Features Target Cancellation (MFTC): An Attentional Visual Conjunction Search Test. Normative Values for the Italian Population. Neurol. Sci. Off. J. Ital. Neurol. Soc. Ital. Soc. Clin. Neurophysiol..

[37] Roid G.H., Miller L.J., Pomplun M., Koch C. (2013). Leiter International Performance Scale.

[38] Demeyere N., Riddoch M.J., Slavkova E.D., Bickerton W.-L., Humphreys G.W. (2015). The Oxford Cognitive Screen (OCS): Validation of a Stroke-Specific Short Cognitive Screening Tool. Psychol. Assess.

[39] Mesulam M.-M. (2000). Principles of Behavioral and Cognitive Neurology.

[40] Weintraub S., Mesulam M.M. (1988). Visual Hemispatial Inattention: Stimulus Parameters and Exploratory Strategies. J. Neurol. Neurosurg. Psychiatry.

[41] Posner M.I. (1980). Orienting of Attention. Q. J. Exp. Psychol..

[42] Castiello U., Umiltà C. (1990). Size of the Attentional Focus and Efficiency of Processing. Acta Psychol..

[43] Chun M.M., Golomb J.D., Turk-Browne N.B. (2011). A Taxonomy of External and Internal Attention. Annu. Rev. Psychol..

[44] He S., Cavanagh P., Intriligator J. (1996). Attentional Resolution and the Locus of Visual Awareness. Nature.

[45] He S., Cavanagh P., Intriligator J. (1997). Attentional Resolution. Trends Cogn. Sci..

[46] Intriligator J., Cavanagh P. (2001). The Spatial Resolution of Visual Attention. Cognit. Psychol..

[47] Scolari M., Kohnen A., Barton B., Awh E. (2007). Spatial Attention, Preview, and Popout: Which Factors Influence Critical Spacing in Crowded Displays?. J. Vis..

[48] Yeshurun Y., Rashal E. (2010). Precueing Attention to the Target Location Diminishes Crowding and Reduces the Critical Distance. J. Vis..

[49] Albonico A., Martelli M., Bricolo E., Frasson E., Daini R. (2018). Focusing and Orienting Spatial Attention Differently Modulate Crowding in Central and Peripheral Vision. J. Vis..

[50] Daini R., Primativo S., Albonico A., Veronelli L., Malaspina M., Corbo M., Martelli M., Arduino L.S. (2021). The Focal Attention Window Size Explains Letter Substitution Errors in Reading. Brain Sci..

[51] Measso G., Cavarzeran F., Zappalà G., Lebowitz B.D., Crook T.H., Pirozzolo F.J., Amaducci L.A., Massari D., Grigoletto F. (1993). The Mini-mental State Examination: Normative Study of an Italian Random Sample. Dev. Neuropsychol..

[52] ISTAT (2021). Demographic Indicators.

[53] Bailey I.L., Lovie-Kitchin J.E. (2013). Visual Acuity Testing. From the Laboratory to the Clinic. Vision Res..

[54] (2017). Ophthalmic Optics—Visual Acuity Testing—Standard and Clinical Optotypes and Their Presentation.

[55] Byrd D.A., Touradji P., Tang M.-X., Manly J.J. (2004). Cancellation Test Performance in African American, Hispanic, and White Elderly. J. Int. Neuropsychol. Soc. JINS.

[56] Deng I.D., Chung L., Talwar N., Tam F., Churchill N.W., Schweizer T.A., Graham S.J. (2019). Functional MRI of Letter Cancellation Task Performance in Older Adults. Front. Hum. Neurosci..

[57] Statsenko Y., Habuza T., Gorkom K.N.-V., Zaki N., Almansoori T.M. (2020). Applying the Inverse Efficiency Score to Visual–Motor Task for Studying Speed-Accuracy Performance While Aging. Front. Aging Neurosci..

[58] Townsend J.T., Ashby F.G. (1983). Stochastic Modeling of Elementary Psychological Processes.

[59] Garzia R.P., Richman J.E., Nicholson S.B., Gaines C.S. (1990). A New Visual-Verbal Saccade Test: The Developmental Eye Movement Test (DEM). J. Am. Optom. Assoc..

[60] Knorr E.M., Ng R.T., Tucakov V. (2000). Distance-Based Outliers: Algorithms and Applications. VLDB J..

[61] Capitani E., Laiacona M. (1997). Composite Neuropsychological Batteries and Demographic Correction: Standardization Based on Equivalent Scores, with a Review of Published Data. The Italian Group for the Neuropsychological Study of Ageing. J. Clin. Exp. Neuropsychol..

[62] Capitani E., Laiacona M. (2017). Outer and Inner Tolerance Limits: Their Usefulness for the Construction of Norms and the Standardization of Neuropsychological Tests. Clin. Neuropsychol..

[63] Heaton R.K., Avitable N., Grant I., Matthews C.G. (1999). Further Crossvalidation of Regression-Based Neuropsychological Norms with an Update for the Boston Naming Test. J. Clin. Exp. Neuropsychol..

[64] Facchin A., Vallar G., Daini R. (2021). The Brentano Illusion Test (BRIT): An Implicit Task of Perceptual Processing for the Assessment of Visual Field Defects in Neglect Patients. Neuropsychol. Rehabil..

[65] Trojano L., Siciliano M., Pedone R., Cristinzio C., Grossi D. (2015). Italian Normative Data for the Battery for Visuospatial Abilities (TERADIC). Neurol. Sci..

[66] Rigoli M., Facchin A., Cardile D., Beschin N., Luzzatti C. (2021). Open-Source Open-Access Reaction Time Test (OORTT): An Easy Tool to Assess Reaction Times. Neurol. Sci..

[67] Siciliano M., Chiorri C., Battini V., Sant’Elia V., Altieri M., Trojano L., Santangelo G. (2019). Regression-Based Normative Data and Equivalent Scores for Trail Making Test (TMT): An Updated Italian Normative Study. Neurol. Sci..

[68] Akaike H., Kotz S., Johnson N.L. (1992). Information Theory and an Extension of the Maximum Likelihood Principle.

[69] Wagenmakers E.-J., Farrell S. (2004). AIC Model Selection Using Akaike Weights. Psychon. Bull. Rev..

[70] Facchin A., Rizzi E., Vezzoli M. (2022). A Rank Subdivision of Equivalent Score for Enhancing Neuropsychological Test Norms. Neurol. Sci..

[71] R Core Team (2022). R: A Language and Environment for Statistical Computing.

[72] Johnston W.A., Dark V.J. (1986). Selective Attention. Annu. Rev. Psychol..

[73] Gómez-Pérez E., Ostrosky-Solís F. (2006). Attention and Memory Evaluation Across the Life Span: Heterogeneous Effects of Age and Education. J. Clin. Exp. Neuropsychol..

[74] Connor C.E., Egeth H.E., Yantis S. (2004). Visual Attention: Bottom-Up Versus Top-Down. Curr. Biol..

[75] Nougier V., Rossi B., Alain C., Taddei F. (1996). Evidence of Strategic Effects in the Modulation of Orienting of Attention. Ergonomics.

[76] Eriksen C.W., St. James J.D. (1986). Visual Attention within and around the Field of Focal Attention: A Zoom Lens Model. Percept. Psychophys..

[77] Rösler A., Mapstone M.E., Hays A.K., Mesulam M.-M., Rademaker A., Gitelman D.R., Weintraub S. (2000). Alterations of Visual Search Strategy in Alzheimer’s Disease and Aging. Neuropsychology.

[78] Solbakk A.-K., Alpert G.F., Furst A.J., Hale L.A., Oga T., Chetty S., Pickard N., Knight R.T. (2008). Altered Prefrontal Function with Aging: Insights into Age-Associated Performance Decline. Brain Res..

[79] Filoteo J.V., Williams B.J., Rilling L.M., Roberts J.W. (1997). Performance of Parkinson’s Disease Patients on the Visual Search and Attention Test: Impairment in Single-Feature but Not Dual-Feature Visual Search. Arch. Clin. Neuropsychol..

[80] Farnè A., Buxbaum L.J., Ferraro M., Frassinetti F., Whyte J., Veramonti T., Angeli V., Coslett H.B., Làdavas E. (2004). Patterns of Spontaneous Recovery of Neglect and Associated Disorders in Acute Right Brain-Damaged Patients. J. Neurol. Neurosurg. Psychiatry.

